# Mortality following elective and emergency colectomy in patients with cirrhosis: a population-based cohort study from England

**DOI:** 10.1007/s00384-021-04061-y

**Published:** 2021-12-11

**Authors:** Alfred Adiamah, Colin J. Crooks, John S. Hammond, Peter Jepsen, Joe West, David J. Humes

**Affiliations:** 1 Nottingham Digestive Diseases Biomedical Research Unit, Nottingham Digestive Diseases Centre, National Institute for Health Research (NIHR) Nottingham Biomedical Research Centre, Queen’s Medical Centre, Nottingham University Hospitals NHS Trust and University of Nottingham, QMC Campus, E Floor West Block, Nottingham, NG7 2UH UK; 2 Department of Hepatobiliary and Transplantation Surgery, Freeman Hospital, Freeman Rd, High Heaton, Newcastle upon Tyne, NE7 7DN UK; 3 Department of Hepatology and Gastroenterology, Aarhus University Hospital, Aarhus, Denmark; 4 Division of Epidemiology and Public Health, School of Medicine, Clinical Sciences Building, University of Nottingham, City Hospital, Nottingham, NG5 1PB UK

**Keywords:** Cirrhosis, Colorectal surgery, Emergency surgery, Postoperative mortality

## Abstract

**Background:**

Patients with cirrhosis undergoing colectomy have a higher risk of postoperative mortality, but contemporary estimates are lacking and data on associated risk and longer term outcomes are limited. This study aimed to quantify the risk of mortality following colectomy by urgency of surgery and stage of cirrhosis.

Data sources.

Linked primary and secondary-care electronic healthcare data from England were used to identify all patients undergoing colectomy from January 2001 to December 2017. These patients were classified by the absence or presence of cirrhosis and severity. Case fatality rates at 90 days and 1 year were calculated, and cox regression was used to estimate the hazard ratio of postoperative mortality controlling for age, gender and co-morbidity.

**Results:**

Of the total, 36,380 patients undergoing colectomy, 248 (0.7%) had liver cirrhosis, and 70% of those had compensated cirrhosis. Following elective colectomy, 90-day case fatality was 4% in those without cirrhosis, 7% in compensated cirrhosis and 10% in decompensated cirrhosis. Following emergency colectomy, 90-day case fatality was higher; it was 16% in those without cirrhosis, 35% in compensated cirrhosis and 41% in decompensated cirrhosis. This corresponded to an adjusted 2.57 fold (95% CI 1.75–3.76) and 3.43 fold (95% CI 2.02–5.83) increased mortality risk in those with compensated and decompensated cirrhosis, respectively. This higher case fatality in patients with cirrhosis persisted at 1 year.

**Conclusion:**

Patients with cirrhosis undergoing emergency colectomy have a higher mortality risk than those undergoing elective colectomy both at 90 days and 1 year. The greatest mortality risk at 90 days was in those with decompensation undergoing emergency surgery.

**Supplementary Information:**

The online version contains supplementary material available at 10.1007/s00384-021-04061-y.

## Introduction

The overall incidence of chronic liver disease and cirrhosis is rising in most European and Western populations [1] secondary to alcohol misuse and the obesity pandemic [2]. These two important modifiable risk factors also contribute to several other gastrointestinal diseases, including benign [3] and malignant colonic diseases [4, 5] for which surgery is often indicated. The case fatality rate at 30-day and 90-day after colectomy, not accounting for age, malignant status and urgency, is of the order of 4.3–8.5% and 9.1–11.3%, respectively [6, 7]. However, colectomy in patients with liver cirrhosis is associated with a further increased risk of operative mortality [8] which correlates with the severity of liver disease and underlying comorbidities [9].

Studies that originated from the 1980s reported that mortality after laparotomy in people with cirrhosis was so high as to preclude surgery in this population [10]. Perioperative and postoperative care pathways along with changes in surgical technique have advanced since then [11] and have led to an uptake of surgery in higher risk groups [12]. However, most contemporary studies directly investigating postoperative outcomes in patients with cirrhosis have been limited to single-centre retrospective observational studies with an inherently higher risk of bias [8, 13, 14]. Additionally, these studies [13–17] have explored only short-term outcomes limited to the in-hospital period or 30 days following surgery and have not focused on the difference between elective and emergency cases [15–17]. A recent systematic review that focused on alcoholic liver disease was able to identify only two studies reporting on mortality risk following colectomy in this patient group, emphasizing the scarcity of work in this area [8].

More recent work, that have defined mortality risk stratification following surgery in patients with cirrhosis, were able to provide granular details on the severity of cirrhosis but could only class procedures into large categories such as abdominal wall, abdominal and vascular surgery but not into the individual procedure types [18]. A recent large population-based study investigated mortality risk in several abdominal surgery which included 860 patients undergoing colorectal resection but could not provide data on whether the procedures were for benign or malignant indications.[19].

Precise estimates of the risk of mortality after elective or emergency colectomy in the short- and longer term periods are inadequately defined in the currently available literature. This population-based cohort study aimed to determine the risk of mortality following colectomy by urgency of the procedure and reporting the 90-day and 1-year mortality risk using linked electronic healthcare data from England.

## Methods

The study was approved by the Independent Scientific Advisory Committee approval board (Protocol 19‐193R).

### Patients and data sources

Linked primary and secondary care electronic healthcare databases which have been previously described in detail were utilized in this study [20–22]. In brief, the Clinical Practice Research Datalink (CPRD) is a primary care database that contains diagnostic and prescription data for approximately 14.1 million active patients. The Hospital Episodes Statistics data (HES) is a prospectively collected statutory record of each episode of an admitted patient care delivered in England, either by NHS hospitals or in the independent sector but NHS commissioned. Death certificate data from the Office for National Statistics (ONS) were used to define mortality.

### Cohort

The cohort of patients aged 18 years and over were identified using OPCS codes for colectomy procedures from HES data between 1st of January 2001 and 31st of December 2017. Operations that were confined to the rectum and anal canal were excluded. Patients were also excluded if they were not in a linked general practice. All patients were followed up until they died, transferred out of a participating general practice, or for 1 year.

### Exposed cohort

Patients with liver cirrhosis were defined by the presence of diagnosis codes in either HES or CPRD at any time point prior to the date of surgery [23]. This included the presence of a Read code for cirrhosis, oesophageal varices and/or portal hypertension in the CPRD data. Similarly the presence of ICD-10 and OPCS-code related to cirrhosis, varices or treatment for varices was used to define cirrhosis in secondary care data. It has been shown previously that more than 90% of patients with a diagnosis in secondary care have supportive evidence of liver cirrhosis, entered in either their death certificate or in their primary care records [24]. The remainder of the patients with no evidence of cirrhosis in their primary or secondary care electronic healthcare records were defined as the unexposed cohort (no cirrhosis group).

### Severity of cirrhosis

The patients with cirrhosis were further sub-classified as being either in a compensated or decompensated disease state at the time of diagnosis using the Baveno IV classification [24, 25]. The Baveno IV classification as a surrogate for laboratory-derived indices of severity of cirrhosis has been validated [26, 27] and used in other population-based studies investigating patients with cirrhosis [24, 28].

### Covariates

The underlying diagnosis for each colectomy was defined as either benign or malignant. The latter, if it was associated with an ICD-10 code for colorectal cancer diagnosis in CPRD and HES data (ICD‐10 sections C18–20, excluding C18·1 Appendix). Benign or non‐malignant diagnoses were confirmed from the ICD‐10 discharge codes associated with the admission and included inflammatory bowel disease (IBD), diverticular disease and other (Supplementary Table 1: supporting information). Age was categorised into three groups: 18–54 years, 55–69 years and 70 years or older. Comorbidity was determined from the CPRD and HES data up to the date of surgery and classified using the Charlson comorbidity index [29] into 0, 1 and ≥ 2. The type of admission was defined as elective or emergency, based on the recorded type of admission associated with the colectomy procedure. The English Index of Multiple Deprivation (IMD2015) measures relative levels of deprivation in 32,844 small areas or neighbourhoods, called Lower Layer Super Output Areas in England with patients classified by their postcodes alone. These scores were categorized into quintiles from 1 to 5 (most to least deprived). Laparoscopic and robotic surgeries were grouped together as minimal access surgery using supplementary OPCS codes.

### Outcomes

The primary outcome of 90-day mortality was defined from linked ONS deaths registration records and included all deaths occurring on the date of surgery and up to 90 days after. The secondary outcomes were total hospital length of stay and mortality at 1-year post-surgery.

### Statistical analysis

The basic characteristics of the cohort were described using frequencies and percentages for categorical variables and medians with their associated interquartile ranges (IQR) as appropriate. The difference in proportion of patients undergoing a colectomy procedure who also had a diagnosis of cirrhosis was examined per year over the study time period using a chi-squared test.

Case fatality at 90 days and 1 year were calculated using standard Kaplan–Meier techniques, to determine the number of deaths and censoring occurring following elective and emergency colectomy. Absolute rates of death (per 1000 person‐years (p-years)) with 95% confidence intervals (95% CI) were calculated by dividing the number of deaths by the person‐time at risk at 90 days after operation. This was done overall and then separately for each covariate of interest. A Cox proportional hazards model was then fitted for 90-day mortality using the following exposures: cirrhosis, age, gender, comorbidity, socioeconomic status and indication for surgery, to estimate the unadjusted and adjusted hazard ratios (HRs) and their respective 95% confidence intervals. The proportional hazards assumption was tested using log–log plots of cumulative hazards.

Given the reported differences in outcomes following emergency and elective surgery [30], it was decided a priori that case fatality and survival will be stratified by urgency of admission for all analyses. All data management and analyses were performed using Stata® version 16 (StataCorp, College Station, TX, USA).

## Results

### Demographics

A total of 36,380 eligible patients were included who underwent an elective or emergency colectomy for benign or malignant indications. Of these, 248 (0.7%) had liver cirrhosis (137 elective (55.2%) and 111 emergency (44.8%)).

There was a male preponderance amongst the patients with cirrhosis undergoing colectomy. These patients with cirrhosis were younger than patients without cirrhosis yet had a greater burden of comorbidity. They also had lower socioeconomic status and were more likely to undergo emergency surgery. In total, 70.6% (175/248) of patients with cirrhosis had compensated disease, and 29.4% (73/248) had decompensated disease. The operative approach was open colectomy for the majority of patients (Table 1).

**Table 1:** Patient demographics

	**Elective surgery**				
	Cirrhosis (*n* = 137)		Non-cirrhosis (*n* = 23,969)		*p*-value
	(*n* =)	%	(*n* =)	%	
Gender					
Male	85	62.04	12,325	51.42	0.013
Female	52	37.96	11,644	48.58	
Age					
18–54	30	21.90	4453	18.58	0.102
55–69	53	38.69	7875	32.85	
≥ 70	54	39.42	11,641	48.57	
Charlson comorbidity					
0	5	3.65	4673	19.50	< 0.0001
1	9	6.57	1890	7.89	
2	123	89.78	17,406	72.62	
Deprivation					
1	24	17.52	5758	24.05	0.003
2	19	13.87	5663	23.65	
3	38	27.74	5281	22.06	
4	29	21.17	4105	17.14	
5	27	19.71	3137	13.10	
Malignancy status					
Benign	52	37.96	7068	29.49	0.030
Cancer	85	62.04	16,901	70.51	
Operative approach					
Minimally invasive	31	22.63	5600	23.36	0.839
Open	106	77.37	18,369	76.64	
Length of stay					
Median (IQR)	12-days	( 8–17 days)	10 days	(7–14 days)	0.0023

	**Emergency surgery**				
	Cirrhosis (*n* = 111)		Non-cirrhosis (*n* = 12,163)		*p*-value
	(*n* =)	%	(*n* =)	%	
Gender					
Male	63	56.76	5571	45.80	0.021
Female	48	43.24	6592	54.20	
Age					
18–54	32	28.83	3284	27.00	0.001
55–69	44	39.64	3108	25.55	
≥ 70	35	31.53	5771	47.45	
Charlson comorbidity					
0	10	9.01	4908	40.35	< 0.0001
1	21	18.92	2187	17.98	
2	80	72.07	5068	41.67	
Deprivation					
1	20	18.02	2572	21.18	0.044
2	22	19.82	2566	21.13	
3	28	25.23	2695	22.20	
4	12	10.81	2236	18.42	
5	29	26.13	2073	17.07	
Malignancy status					
Benign	83	74.77	7888	64.85	0.030
Cancer	28	25.23	4275	35.15	
Operative approach					
Minimally invasive	4	3.60	484	3.98	0.840
Open	107	96.40	11,679	96.40	
Length of stay					
Median (IQR)	15 days	(9–27)	15 days	(10–26)	0.5704

The indication for colectomy differed between urgency of surgery. In elective surgery, the indication was cancer in 62% of patients with cirrhosis and 70% of patients without cirrhosis. Contrastingly, emergency colectomy was for benign indications in 75% of patients with cirrhosis and 65% of patients without cirrhosis (Supplementary Table 1).

The median length of stay differed between patients with cirrhosis and those without cirrhosis following elective (12 (8–17) days vs 10 (7–14) days, *p* = 0.0023) but not emergency colectomy (15 (9–27) days vs 15 (10–26) days, *p* = 0.5704) (Table 1).

### Trend of proportion of patients with cirrhosis who underwent colectomy

The proportion of patients undergoing a colectomy who had a diagnosis of cirrhosis increased from 0.40% in 2001 to 1.07% by the end of 2017 (*χ*^2^ (16, *N* = 36,380) = 50.53, *p* < 0.0001), representing 1 in every 100 colectomies. The increase in the proportion of patients undergoing colectomy who had cirrhosis was statistically significant in elective colectomy (*χ*^2^ (16, *N* = 24,106) = 61.04, *p* < 0.0001), (*χ*^2^ *p* < 0.0001) but not in those undergoing emergency colectomy over the same time period (*χ*^2^ (16, *N* = 12,274) = 18.67, *p* = 0.286).

### Case fatality at 90 days

The 90-day case fatality varied by urgency of surgery. Following elective colectomy, 90-day case fatality was 4% in patients without cirrhosis, 7% in those with compensated cirrhosis and 10% in those with decompensated cirrhosis. However, after emergency colectomy, 90-day case fatality was higher in all categories (Table 2). Patients with decompensated cirrhosis had a higher case fatality in both emergency and elective settings compared with patients without cirrhosis. However, the case fatality difference was the highest following emergency colectomy (absolute difference of 25%) (Table 2).

**Table 2:** The 90-day and 1-year case fatality in patients with and without cirrhosis undergoing elective and emergency colectomy

	**90-day case fatality** **% (95% CI)**	**Case fatality difference**	**I-year case fatality** **% (95% CI)**	**Case fatality difference**
**Elective colectomy**				
No cirrhosis	3.91 (3.67–4.16)	-	9.37 (9.00–9.75)	
Compensated cirrhosis	7.14 (2.92–14.16)	3.23 (− 1.87–8.33)	16.33 (9.63–25.16)	6.96 (− 0.37–14.28)-
Decompensated cirrhosis	10.26 (2.87–24.22)	6.35 (− 3.18–15.87)	15.38 (5.86–30.52)	6.01 (− 5.3–17.34)
**Emergency colectomy**				
No cirrhosis	16.04 (15.39–16.70)	-	24.66 (23.89–25.43)	-
Compensated cirrhosis	35.06 (24.53–46.78)	19.02 (8.35–29.70)	46.75 (35.29–58.48)	22.10 (10.93–33.27)
Decompensated cirrhosis	41.18 (24.65–59.30)	25.14(8.58–41.69)	41.18 (24.65–59.30)	16.52 (0.04–33.08)

Legend

The case fatality difference is the difference in fatality between the either compensated or decompensated cirrhosis and the no cirrhosis group

**Fig. 1:**
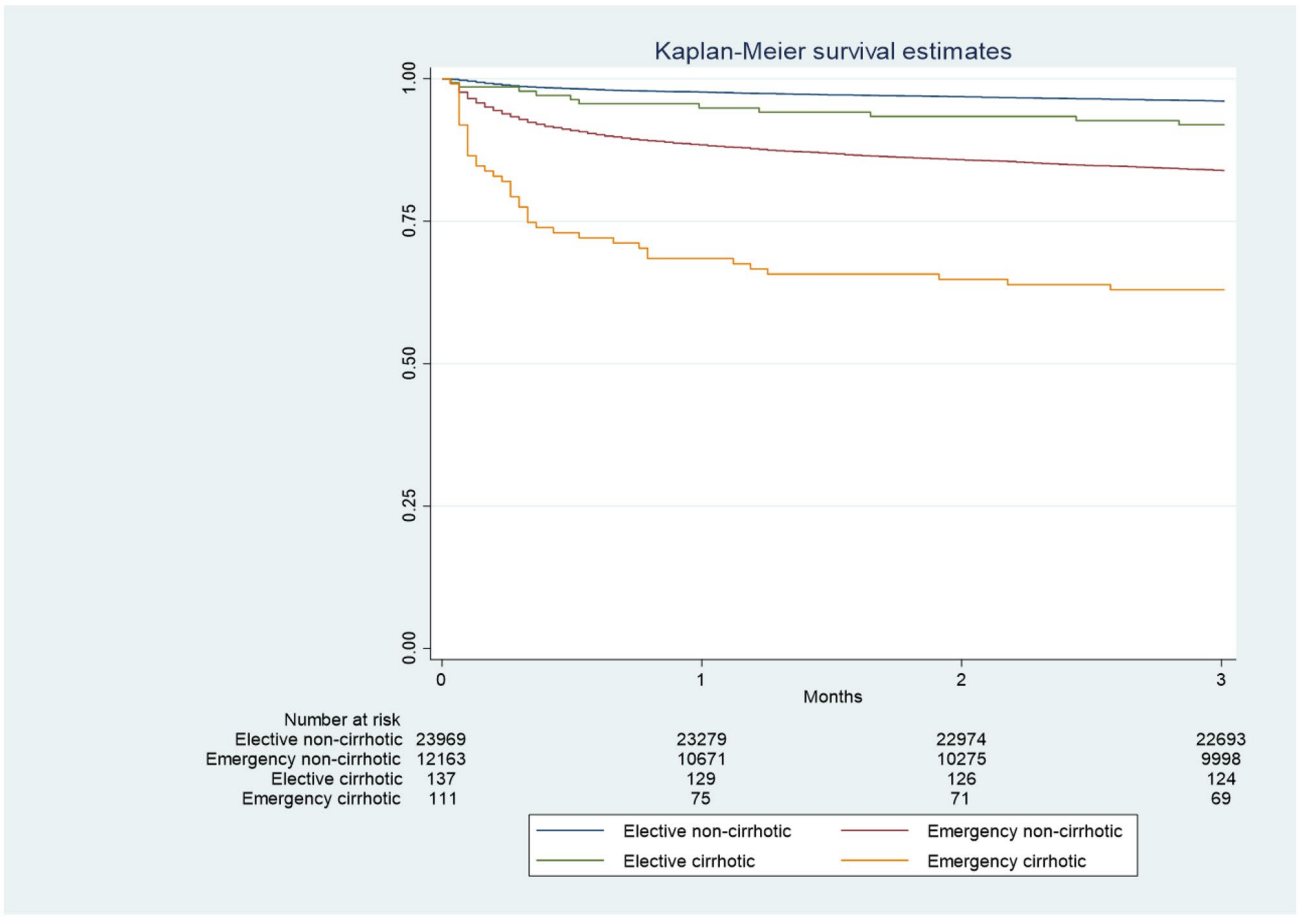
Ninety-day survival in cases and controls by method of admission

The Kaplan–Meier survival curves (Fig. 1) demonstrated that 90-day survival was worse for patients with cirrhosis undergoing elective and emergency surgery compared to those without cirrhosis. The survival curves by severity of cirrhosis (supplementary Fig. 1) demonstrated the higher risk of mortality in those with decompensated disease.

### Multivariable modelling of mortality in patients with cirrhosis vs those without cirrhosis at 90 days

The absolute rates of death following elective colectomy in patients without cirrhosis was 162 per 1000 p-years compared with 306 per 1000 p-years in those with compensated cirrhosis and 436 per 1000 p-years in decompensated cirrhosis (Table 3). This corresponded to an adjusted 1.7 fold increase in mortality at 90 days following colectomy in patients with compensated cirrhosis compared with those without cirrhosis (adjusted HR 1.7, 95% CI 0.81–3.58). Following elective colectomy in those with decompensated cirrhosis, there was an adjusted 2.8-fold increase in mortality at 90 days when compared to those without cirrhosis (adjusted HR 2.79 (95% CI 1.04–7.46). In those undergoing emergency colectomy, those with compensated cirrhosis had an adjusted 2.6 fold (adjusted HR 2.57 (1.75–3.76) increase in mortality at 90 days, whereas those with decompensated cirrhosis had an adjusted 3.4-fold (adjust HR 3.43 (95% CI 2.02–5.83) increase in mortality at 90 days when compared with patients without cirrhosis (Table 3 and Table 4).

**Table 3:** The 90-day rates and hazard ratio of death in cirrhotics and non-cirrhotics undergoing elective surgery

	**Absolute rates**		**Unadjusted HR**		Adjusted HR*	
	**Rate (per 1000 p-yrs)**	**95% CI**	**HR**	**95% CI**	**HR**	**95% CI**
**Cohort**						
Non-cirrhosis	161.84	151.80–172.54	1.0 (ref)		1.0 (ref)	
Compensated cirrhosis	306.35	146.05–642.60	1.87	0.89–3.94	1.70	0.81–3.58
Decompensated cirrhosis	436.13	163.69–1162.02	2.68	1.00–7.16	2.79	1.04–7.46
**Gender**						
Male	191.73	176.63–208.12	1.0 (ref)		1.0 (ref)	
Female	132.57	119.85–146.66	0.69	0.61–0.79	0.68	0.60–0.78
**Age (years)**						
18–54	33.55	24.31–46.31	1.0 (ref)		1.0 (ref)	
55–69	100.76	87.57–115.94	3.00	2.11–4.26	2.93	2.05–4.20
≥ 70	257.21	239.05–276.76	7.60	5.46–10.58	7.69	5.46–10.84
**No. of comorbidities**						
0	75.97	61.57–93.73	1.0 (ref)		1.0 (ref)	
1	162.17	129.13–203.66	2.13	1.56–2.90	1.71	1.25–2.34
≥ 2	186.48	173.90–199.98	2.44	1.96–3.05	1.69	1.33–2.15
**IMD**						
1	130.21	112.65–150.52	1.0 (ref)		1.0 (ref)	
2	157.50	137.84–179.97	1.21	0.99–1.47	1.20	0.99–1.47
3	156.11	135.95–179.26	1.20	0.98–1.46	1.20	0.99–1.25
4	192.11	166.71–221.39	1.47	1.20–1.80	1.53	1.25–1.87
5	206.05	176.12–241.05	1.58	1.27–1.95	1.71	1.382.12
**Indication**						
Non‐malignant	128.76	112.89–146.87	1.0 (ref)		1.0 (ref)	
Malignant	177.17	164.74–190.54	1.37	1.18–1.60	0.72	0.60–0.85

**Table 4:** The 90-day rates and hazard ratio of death in cirrhotics and non-cirrhotics undergoing emergency surgery

	**Absolute rates**		**Unadjusted HR**		Adjusted HR*	
	**Rate (per 1000 p-yrs)**	**95% CI**	**HR**	**95% CI**	**HR**	**95% CI**
**Cohort**						
Non-cirrhosis	737.97	705.94–771.45	1.0 (ref)		1.0 (ref)	
Compensated cirrhosis	1979.75	1357.68–2886.85	2.48	1.70–3.62	2.57	1.75–3.76
Decompensated cirrhosis	2671.28	1582.07–4510.38	3.36	1.98–5.68	3.43	2.02–5.83
**Gender**						
Male	670.27	626.18–717.45	1.0 (ref)		1.0 (ref)	
Female	815.84	770.25–864.12	1.21	1.11–1.32	1.08	0.99–1.18
**Age (years)**						
18–54	139.10	115.39–167.68	1.0 (ref)		1.0 (ref)	
55–69	585.41	531.65–644.61	4.13	3.35–5.10	3.82	3.09–4.72
≥ 70	1260.51	1197.65–1326.67	8.55	7.04–10.38	7.72	6.32–9.44
**No. of comorbidities**						
0	410.62	374.83–449.82	1.0 (ref)		1.0 (ref)	
1	771.98	696.80–855.26	1.84	1.60–2.11	1.39	1.21–1.59
≥ 2	1094.63	1033.52–1159.36	2.57	2.31–2.86	1.66	1.48–1.85
**IMD**						
1	705.40	639.71–777.84	1.0 (ref)		1.0 (ref)	
2	695.22	629.85–767.37	0.98	0.86–1.13	0.98	0.85–1.12
3	728.49	662.79–800.71	1.03	0.90–1.18	1.02	0.89–1.17
4	820.38	743.34–905.41	1.15	1.00–1.33	1.20	1.04–1.38
5	823.60	743.82–911.95	1.16	1.01–1.34	1.26	1.09–1.45
**Indication**						
Non‐malignant	699.70	661.36–740.26	1.0 (ref)		1.0 (ref)	
Malignant	837.89	781.17–898.72	1.19	1.09–1.30	0.80	0.73–0.87

### Case fatality at 1 year

The case fatality at 1 year varied by urgency of surgery but not by the severity of cirrhosis (Table 2).

### Multivariable modelling of mortality in patients with cirrhosis vs those without cirrhosis at 1 year

The absolute rates of death following elective colectomy in patients without cirrhosis at 1 year was 102.28 per 1000 p-years compared with 186.36 per 1000 p-years in patients with cirrhosis. This corresponded to an adjusted 1.7 fold increase in mortality at 1 year in patients with cirrhosis (adjusted HR 1.72, 95% CI 1.13–2.62) (Supplementary Table 2). In those undergoing emergency colectomy, the absolute mortality risk was 314.04 per 1000 p-years in those without cirrhosis and 785.22 per 1000 p-years in those with cirrhosis, and this corresponded to an adjusted 2.38 fold (adjusted HR 2.57 (1.75–3.76) increase in mortality at 1 year (Supplementary Table 3).

## Discussion

### What this study found

There was an increase in the proportion of people undergoing colectomy who had liver cirrhosis over the study period such that in 2017, 1 in every 100 patients undergoing colectomy had cirrhosis. Overall, cirrhosis was associated with a substantially higher risk of postoperative mortality in both elective and emergency surgery when compared with those without cirrhosis. However, within the patient group with cirrhosis, the case fatality at 90 days following elective colectomy of 7% in compensated cirrhosis and 10% in decompensated cirrhosis was substantially less than 35% and 41%, respectively, following emergency colectomy. For all comparable categories, patients with cirrhosis had a higher risk of postoperative mortality than those without cirrhosis. This higher risk of mortality than patients without cirrhosis persisted at the 1-year follow-up.

### What is already known

Liver disease in general and cirrhosis in particular is increasing in incidence and prevalence in the UK. However, it was not known if the background increase in population prevalence of cirrhosis was reflected in the number of patients with cirrhosis undergoing surgery. A Danish population-based study on colorectal cancer surgery using data from 1996 to 2009 reported that 0.4% of their total cohort of 39,840 patients had liver cirrhosis [15]. Another study that used American College of Surgeons National in-patient data covering 1998 to 2005 found a 0.8% prevalence of cirrhosis in their cohort of half-a-million patients undergoing colorectal procedures [17]. Our study, using a more contemporary cohort, has found an overall 0.7% prevalence of cirrhosis in patients undergoing colectomy over the 17-year duration of the study. At the end of the study period (2017), the finding that 1.07% of patients undergoing colectomy had cirrhosis suggests that the increasing prevalence of the disease is being reflected in the numbers undergoing surgery. Another explanation for this finding is that advancements in perioperative and postoperative care is allowing more high-risk cases, which would have been previously been precluded from surgical intervention to be considered for surgery.

Cirrhosis is generally acknowledged to be associated with a higher risk of postoperative complications and mortality. However, the magnitude of the mortality risk, especially the longer-term mortality risk after colectomy, has not been defined using a population-based cohort. Previous population-based studies have reported either in-hospital or 30-day mortality [15–17], with medium- and long-term mortality risk completely unaddressed. The most recent and largest cohort of patients with cirrhosis undergoing abdominal surgery could also only provide short-term mortality risk and could not define specific procedure types or indication [18]. Visser et al. [7] argue that 30-day mortality significantly underreports the true risk of death after colectomy. Our study addresses this gap in knowledge in the risk of mortality following colectomy and has shown that mortality risk following surgery in patients with cirrhosis is higher than in those without cirrhosis at 90 days and 1 year. It has shown that whilst those patients with compensated cirrhosis fare better at 90 days, by the 1-year follow-up period, there is no difference in risk by the severity of the disease.

Our results confirmed higher mortality among patients admitted for emergency colectomy compared with those admitted electively at 90 days. This is congruent with results reported from other nationwide [15] and single-centre studies [13, 31] Meunier et al. [13] suggested in their retrospective analysis that the need for emergency surgery in their cohort of patients with cirrhosis was usually for peritonitis and obstruction, with these patients requiring more extensive resections and enduring more postoperative complications. In our analysis, the majority of emergency colectomy procedures (75%) were for non-malignant indications. However, these patients had the highest mortality at 90 days, suggesting the acute presentation and lack of preoperative optimization and planning, potentially contribute to the higher early mortality risk in this group.

### Limitations

This study is one of the largest dataset reported to date evaluating outcomes in patients with cirrhosis undergoing colectomy for benign and malignant indications. It has demonstrated a statistically significant increase in the proportion of people undergoing colectomy who had liver cirrhosis over the study period. If the rising trend of liver disease and cirrhosis persists, then the expectation is more patients with cirrhosis will undergo colectomy procedures in the future, and this study therefore provides a needed and timely understanding of their mortality risk. The patients with and without cirrhosis were drawn from a nationwide database that is representative of the population in England, which makes the results more robust as opposed to single-centre experience.

Nonetheless, a recurring limitation, pertinent to all population-based studies, is the confidence in case definitions as we are dependent on the accuracy of coding of procedures, events and associated morbidity. To overcome this, we ensured that our case definition of colectomy was supported by both an OPCS Code for colectomy and an event date of the operative procedure. Additionally, we used a validated algorithm to define cirrhosis within HES and CPRD data which has been shown to have over 90% concordance when validated against patient notes [24]. This provides the confidence and reliability of both our case definition of colectomy and our exposure of cirrhosis.

The MELD and Child–Pugh scores have been used in previous analysis to explore the importance of severity of cirrhosis on mortality risk. However, due to the absence of requisite biochemical and clinical data, these scores could not be computed for our analysis. To overcome this, we utilized the Baveno IV classification [24] of compensated and decompensated cirrhosis which relies on the presence or absence of ascites, varices and variceal bleeding and has been validated in multiple other studies as sufficiently discriminatory of severity of cirrhosis.

## Conclusion

This study has determined the risk of mortality and survival estimates after elective and emergency colectomy in patients with cirrhosis using a contemporary cohort. It has shown an increase in the proportion of patients undergoing colectomy who had liver cirrhosis, such that in 2017, 1 in every 100 patients undergoing colectomy had cirrhosis. Importantly, it has shown that emergency colectomy in this patient group is associated with a significantly higher risk of mortality than elective procedures. These results should provide surgeons up-to-date information to aid preoperative risk stratification of patients with cirrhosis requiring colectomy.

## Supplementary Information

Below is the link to the electronic supplementary material.

Supplementary file1 (DOCX 19 KB)

Supplementary file2 (PDF 248 KB)

## Declarations

### Author statement

All authors had access to the data and contributed to the drafting of the paper.

### Guarantor of the article

D.J. Humes.
