# Supplementary material for: Mortality following elective and emergency colectomy in patients with cirrhosis: a population-based cohort study from England
Source: Int J Colorectal Dis. 2021 Dec 11;37(3):607–16. doi: 10.1007/s00384-021-04061-y (PMC8885503; doi:10.1007/s00384-021-04061-y)
Supplement: Supplementary file 1 — Supplementary file1 (DOCX 19 KB) [file 384_2021_4061_MOESM1_ESM.docx]

Supplementary Table 1: Indications for surgery in cirrhotics and non-cirrhotics

|  | **Non-cirrhotic**  **(n=36132)** | **Compensated cirrhosis**  **(n=175)** | **Decompensated Cirrhosis**  **(n=73)** |
| --- | --- | --- | --- |
| **Colorectal cancer** | 21176 (58.61%) | 87 (49.71) | 26 (35.62%) |
| **IBD** | 4534 (12.54%) | 26 (14.86%) | 14 (19.18%) |
| **Diverticular disease** | 5708 (15.80%) | 37 (21.14) | 18 (24.66%) |
| **Other*** | 4714 (13.04%) | 25 (14.29) | 15 (20.55%) |

Legend:

*IBD: inflammatory bowel disease

Supplementary Table 2: The 1-year rates and Hazard risk of death in cirrhotics and non-cirrhotics undergoing elective surgery

|  | **Absolute Rates** | | **Unadjusted HR** | | **Adjusted HR**[*****](https://bjssjournals.onlinelibrary.wiley.com/doi/full/10.1002/bjs.9923#bjs9923-note-0003_20) | |
| --- | --- | --- | --- | --- | --- | --- |
|  | **Rate (per 1000 p-yrs)** | **95 % CI** | **HR** | **95% CI** | **HR** | **95% CI** |
| **Cohort** |  |  |  |  |  |  |
| Non-cirrhosis | 102.28 | 98.14-106.60 | 1.0 (ref) |  | 1.0 (ref) |  |
| Cirrhosis | 186.36 | 122.71-283.02 | 1.80 | 1.19-2.75 | 1.72 | 1.13-2.62 |
| **Gender** |  |  |  |  |  |  |
| Female | 112.04 | 106.03-118.39 | 1.0 (ref) |  | 1.0 (ref) |  |
| Male | 93.02 | 87.44-98.95 | 0.83 | 0.77-0.90 | 0.86 | 0.79-0.94 |
| **Age (years)** |  |  |  |  |  |  |
| 18-54 | 27.78 | 23.19-33.27 | 1.0 (ref) |  | 1.0 (ref) |  |
| 55-69 | 74.02 | 68.07-80.50 | 2.66 | 2.18-3.25 | 2.23 | 1.82-2.74 |
| ≥70 | 153.44 | 146.11-161.14 | 5.48 | 4.54-6.60 | 4.51 | 3.71-5.49 |
| **No. of co‐morbidities** |  |  |  |  |  |  |
| 0 | 45.97 | 40.06-52.74 | 1.0 (ref) |  | 1.0 (ref) |  |
| 1 | 78.38 | 66.30-92.67 | 1.70 | 1.37-2.11 | 1.46 | 1.17-1.81 |
| ≥2 | 121.16 | 115.87-126.69 | 2.62 | 2.27-3.03 | 1.78 | 1.52-2.08 |
| **IMD** |  |  |  |  |  |  |
| 1 | 91.13 | 83.39-99.60 | 1.0 (ref) |  | 1.0 (ref) |  |
| 2 | 101.80 | 93.48-110.87 | 1.12 | 0.99-1.26 | 1.10 | 0.98-1.25 |
| 3 | 98.61 | 90.20-107.81 | 1.08 | 0.95-1.23 | 1.07 | 0.94-1.21 |
| 4 | 114.92 | 104.58-126.29 | 1.26 | 1.11-1.43 | 1.28 | 1.12-1.46 |
| 5 | 117.22 | 105.34-130.45 | 1.28 | 1.12-1.47 | 1.35 | 1.17-1.55 |
| **Indication** |  |  |  |  |  |  |
| Non‐malignant | 65.96 | 60.04-72.45 | 1.0 (ref) |  | 1.0 (ref) |  |
| Malignant | 118.39 | 113.10-123.94 | 1.79 | 1.61-1.99 | 1.01 | 0.90-1.14 |
| **Operative Approach** |  |  |  |  |  |  |
| Minimally invasive | 72.06 | 64.90-80.01 | 1.0 (ref) |  | 1.0 (ref) |  |
| Open | 111.41 | 106.53-116.51 | 2.02 | 1.56-2.61 | 1.54 | 1.38-1.73 |

Supplementary Table 3: The 1-year rates and Hazard ratio of death in cirrhotics and non-cirrhotics undergoing emergency surgery

|  | **Absolute rates** | | **Unadjusted HR** | | **Adjusted HR**[*****](https://bjssjournals.onlinelibrary.wiley.com/doi/full/10.1002/bjs.9923#bjs9923-note-0003_20) | |
| --- | --- | --- | --- | --- | --- | --- |
|  | **Rate (per 1000 p-yrs)** | **95 % CI** | **HR** | **95% CI** | **HR** | **95% CI** |
| **Cohort** |  |  |  |  |  |  |
| Non-cirrhosis | 314.04 | 303.00-325.48 | 1.0 (ref) |  | 1.0 (ref) |  |
| Cirrhosis | 785.22 | 595.13-1036.03 | 2.28 | 1.73-3.02 | 2.38 | 1.79-3.15 |
| **Gender** |  |  |  |  |  |  |
| Female | 294.23 | 278.76-310.55 | 1.0 (ref) |  | 1.0 (ref) |  |
| Male | 337.15 | 321.64-353.40 | 1.14 | 1.06-1.22 | 1.06 | 0.99-1.14 |
| **Age (years)** |  |  |  |  |  |  |
| 18-54 | 70.01 | 61.14-80.18 | 1.0 (ref) |  | 1.0 (ref) |  |
| 55-69 | 248.72 | 248.72-288.74 | 3.71 | 3.18-4.34 | 3.12 | 2.67-3.65 |
| ≥70 | 530.42 | 508.46-553.32 | 7.00 | 6.07-8.06 | 5.52 | 4.76-6.40 |
| **No. of co‐morbidities** |  |  |  |  |  |  |
| 0 | 176.67 | 164.37-189.89 | 1.0 (ref) |  | 1.0 (ref) |  |
| 1 | 284.33 | 260.35-310.51 | 1.58 | 1.41-1.77 | 1.26 | 1.12-1.42 |
| ≥2 | 491.45 | 469.36-514.58 | 2.62 | 2.41-2.86 | 1.67 | 1.53-1.82 |
| **IMD** |  |  |  |  |  |  |
| 1 | 301.54 | 278.77-326.17 | 1.0 (ref) |  | 1.0 (ref) |  |
| 2 | 297.48 | 274.82-322.01 | 0.99 | 0.88-1.10 | 0.98 | 0.87-1.09 |
| 3 | 305.23 | 282.69-329.56 | 1.01 | 0.90-1.13 | 1.00 | 0.90-1.12 |
| 4 | 358.42 | 331.18-387.89 | 1.17 | 1.05-1.31 | 1.22 | 1.09-1.36 |
| 5 | 334.41 | 307.38-363.81 | 1.10 | 0.98-1.24 | 1.19 | 1.06-1.33 |
| **Indication** |  |  |  |  |  |  |
| Non‐malignant | 254.12 | 242.03-266.82 | 1.0 (ref) |  | 1.0 (ref) |  |
| Malignant | 440.57 | 418.33-463.99 | 1.68 | 1.56-1.80 | 1.12 | 1.04-1.21 |
| **Operative Approach** |  |  |  |  |  |  |
| Minimally invasive | 157.84 | 122.29-203.72 | 1.0 (ref) |  | 1.0 (ref) |  |
| Open | 323.61 | 312.21-335.42 | 2.02 | 1.56-2.61 | 1.67 | 1.29-2.17 |
