# Supplementary figures and images for: Mortality following elective and emergency colectomy in patients with cirrhosis: a population-based cohort study from England
Source: Int J Colorectal Dis. 2021 Dec 11;37(3):607–16. doi: 10.1007/s00384-021-04061-y (PMC8885503; doi:10.1007/s00384-021-04061-y)

Supplementary Figure 1: 90-day survival by severity of cirrhosis

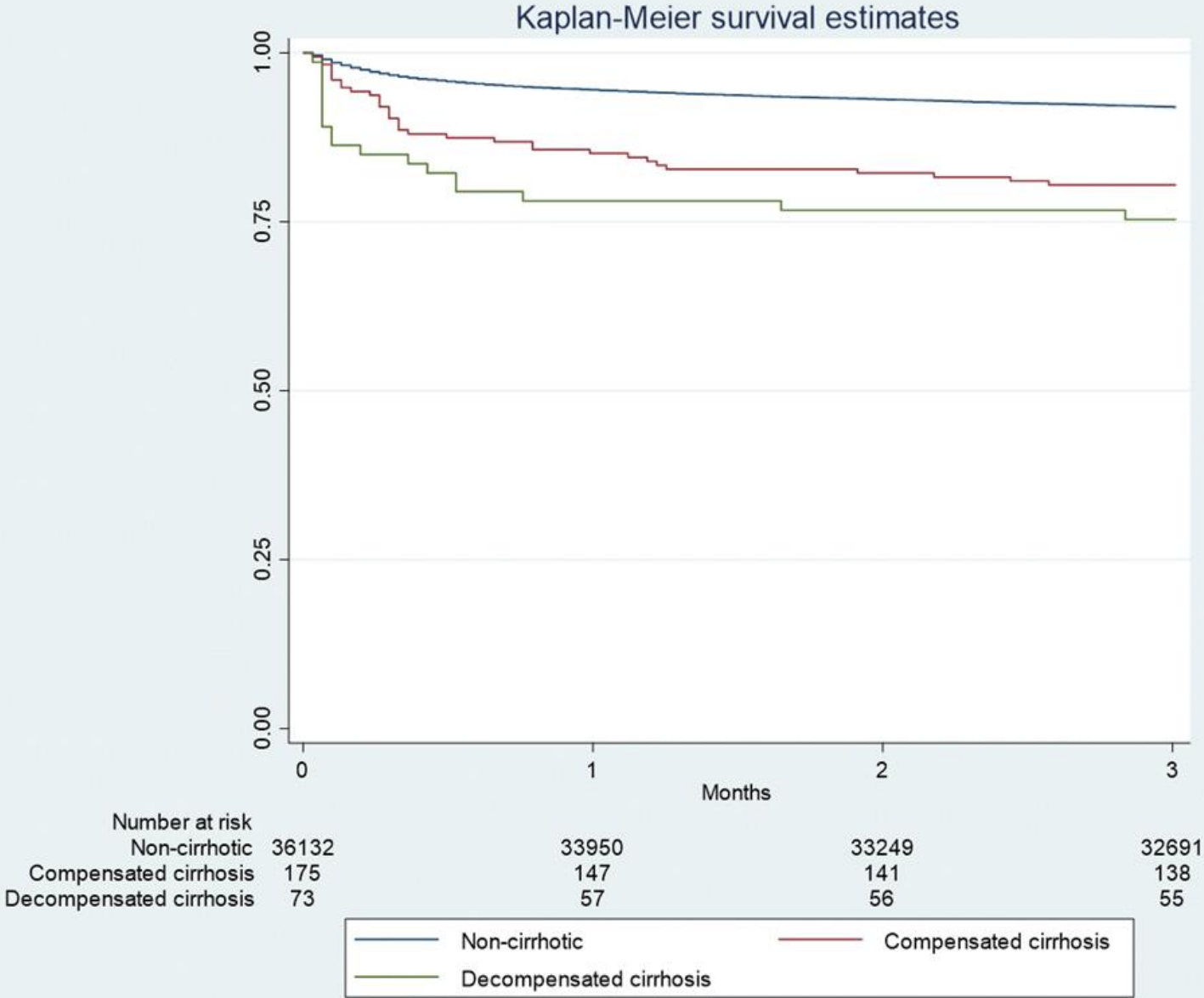

Supplement: Supplementary file 2 — Supplementary file2 (PDF 248 KB) [file 384_2021_4061_MOESM2_ESM.pdf]
